# 局部热消融在NSCLC中的临床应用现状与挑战

**DOI:** 10.3779/j.issn.1009-3419.2020.02.06

**Published:** 2020-02-20

**Authors:** 庆 苟, 泽健 周, 明芳 赵, 晓明 陈, 清 周

**Affiliations:** 1 510080 广州，广东省人民医院（广东省医学科学院）肿瘤介入治疗科 Department of Interventional Oncology, Guangdong Provincial People's Hospital (Guangdong Academy of Medical Sciences), Guangzhou 510080, China; 2 110001 沈阳，中国医科大学附属第一医院 肿瘤内科 The First Hospital of China Medical University, Department of Medical Oncology, Shenyang 110001, China; 3 510080 广州，广东省人民医院（广东省医学科学院），广东省肺癌研究所 Guangdong Provincial People's Hospital (Guangdong Academy of Medical Sciences), Guangdong Lung Cancer Institute, Guangzhou 510080, China

**Keywords:** 肺肿瘤, 热消融, 局部治疗, Lung neoplasms, Thermal ablation, Local treatment

## Abstract

非小细胞肺癌（non-small cell lung cancer, NSCLC）是原发性肺癌的主要类型，手术切除、放化疗、靶向治疗及免疫治疗等是其主要的治疗模式。近年来，热消融在各期NSCLC的临床应用中受到越来越多的关注。热消融作为一种安全、高效的局部治疗手段，合理的临床应用可以给NSCLC患者带来更多的临床获益。然而其在NSCLC中的应用仍然存在许多尚待研究探讨的问题。本文对近年来热消融在NSCLC中的应用现状作以综述，旨在探讨目前存在的争议问题和未来研究方向。

## Abstract (en)

Non-small cell lung cancer (NSCLC) is the most common pathological type of primary lung cancer. Currently, main treatment approaches for NSCLC patients include surgical resection, radiotherapy, chemotherapy, targeted therapy and so on. In recent years, thermal ablation has received increasing attention in the treatment of various stages of NSCLC. As a safe and efficient local treatment, thermal ablation may bring potential clinical benefits to NSCLC patients. However, many issues remain unsolved and further investigation is needed in the clinical application of thermal ablation in NSCLC. In this review, we aim to summarize the applications of thermal ablation in NSCLC and further discuss the emerging controversies as well as future research directions.

肺癌发病率、死亡率均高居前列^[1]^。其中非小细胞肺癌（non-small cell lung cancer, NSCLC）是原发性肺癌的主要类型，约占85%^[2]^。虽然近年来分子靶向治疗、免疫治疗在NSCLC中取得了里程碑式进展，但对于特殊人群或疾病发展特定阶段，有效的局部治疗干预，可能给患者带来更佳的临床获益。热消融（thermal ablation, TAB）作为一种局部治疗，对原发性和转移肺癌均有高效局部杀伤作用，且具有对周围正常组织损伤小、术后恢复快等优势^[3]^。根据产热原理不同，热消融主要分为射频消融（radiofrequency ablation, RFA）和微波消融（microwave ablation, MWA）^[4]^。热消融为NSCLC治疗提供了新的视角，但目前热消融在NSCLC中的应用仍然有待进一步探索和研究，本文就热消融在不同分期NSCLC中的应用现状、存在的争议和挑战等问题进行综述。

## 1 热消融在早期NSCLC中的应用

热消融应用于早期NSCLC（Ⅰa期、Ⅰb期），临床疗效及安全性良好。早期一项多中心、前瞻性研究（RAPTURE）^[5]^报道了RFA治疗Ⅰ期NSCLC的疗效和安全性，结果显示：2年生存（overall survival, OS）率和肿瘤特异性生存（cancer specific survival, CSC）率分别可达75%和92%，最常见的不良反应为气胸和胸腔积液。另一项研究^[6]^进一步肯定了热消融在Ⅰ期NSCLC中的应用价值，热消融治疗后2年和3年生存率可分别达到74%和68%，且无严重并发症。

因全身合并疾病或其他因素，不适合外科手术的早期NSCLC，热消融可以作为一种潜在的有效治疗措施。热消融应用于早期不适合手术的NSCLC（表 1），1年、3年局部控制率可分别达到86.0%-96.0%、64.0%-77.8%，中位OS达30-57个月，1年、2年、3年和5年OS率分别为81.0%-96.0%、63.0%-86.5%、40.0%-67.1%和16.0%-36.3%^[7-14]^。一项比较RFA和立体定向放射治疗（stereotactic body radiotherapy, SBRT）不可手术Ⅰ期NSCLC的系统评价及合并分析显示：热消融1年、2年和3年OS率接近SBRT，同时消融治疗组和SBRT治疗组OS无显著差异（*P* > 0.05），但局部控制率方面，SBRT优于热消融^[15]^。美国国家癌症数据库的一项研究对比了SBRT及热消融治疗Ⅰ期NSCLC的临床疗效，倾向匹配分析显示两组OS无明显差异（*P*=0.06），热消融治疗组1年、2年、3年和5年OS率（85.4%、65.2%、47.8%和24.6%）与SBRT组（86.3%、64.5%、45.9%和26.1%）相比，同样无统计学差异（*P*=0.76, 0.43, 0.32, 0.81）^[16]^。热消融还有可反复多次治疗的优点，Yang等^[17]^对早期NSCLC经MWA治疗后局部复发的患者，再次行MWA治疗，局部控制率高达87.5%，1年、2年、3年和5年OS率分别达100%、74.6%、60.6%和27.0%。

**1:** 热消融治疗不适合外科手术的早期NSCLC Thermal ablation for inoperable patients with early stage NCSLC

Author (yr)	*N*	Mean age (yr)	Stage	Size of tumor (cm)	follow-up time (mo)	Ablation method	Median OS (M)	OS					AE		
								1-yr	3-yr	4-yr	5-yr		Pneumothorax	Pleural effusion	Hemoptysis
Huang **et al** (2018)	50	73	Ⅰa	2.2 (1.0-3.0)	46.9 (8-136)	RFA	47.0	96.0%	67.1%	NA	36.3%		12.0%	8%	NA
Palussière *et al* (2018)	42	71.7	Ⅰa/Ⅰb	2.1 (1.3–3.7)	36	RFA	NA	91.6%	58.3%	NA	NA		12.0%	8.0%	NA
Narsule *et al* (2017）	31	69	Ⅰa	1.88 (0.8-3.0)	45	RFA/MWA	39.0	81.0%	52%	NA	NA		57.1%	NA	NA
Dupuy *et al* (2015)	54	76	Ⅰa	2.1 (0.8-3.0)	24	RFA	NA	86.3%	NA	NA	NA		3% > 3	2% > 3	NA
Xia *et al* (2014)	47	69.4	Ⅰa/Ⅰb	24 > 3.5/23≤3.5	30（7-70）	MWA	NA	89.0%	43.0%	NA	16.0%		63.8%	34.0%	0%
Liu *et al* (2015)	29	78	Ⅰa/Ⅰb	3.1 (1.5-4.8)	25	RFA	57.0	90.5%	65.5%	NA	NA		27.0%	NA	NA
Ambrogi *et al* (2011)	57	74	Ⅰa/Ⅰb	2.6 (1.1-5.0)	47	RFA	33.4	83.0%	40.0%	NA	25.0%		11.0%	6.0%	4.0%
Lanuti *et al* (2009)	31	70	Ⅰ	2.0±1.0	17±11	RFA	30.0	NA	NA	47.0%	NA		13.0%	21.0%	NA
NSCLC: non-small cell lung cancer; RFA: radiofrequency ablation; MWA: microwave ablation; OS: overall survival; NA: not available; M: month.															

对于高龄、基础肺功能差或心功能不全的早期NSCLC，热消融同样安全有效。一项MWA治疗高龄合并心肺功能不全的Ⅰa期或Ⅰb期（T1-2N0M0）NSCLC的研究结果显示：1年、3年、5年局部控制率分别可达96%、64%和48%，中位CSC和中位OS分别为47.7个月和33.8个月，1年、2年、3年和5年OS率分别达89%、63%、43%和16%^[9]^。热消融对肺功能影响甚小，在ACOSOG Z4033研究中，RFA治疗后3个月及24个月第1秒用力呼气容积（forced expiratory volume in 1 second, FEV_1_）和肺弥散功能（carbon monoxide diffusing capacity of lung, DLCO）与基线值相比，无明显降低，相反术后3个月、24个月用力肺活量（forced vital capacity, FVC）较热消融治疗前提高（*P* < 0.05）^[12]^。另一项研究显示：热消融治疗前FVC为1.7 L-4.3 L（平均2.6 L; 中位2.3 L），治疗后为1.6 L-4.9 L（平均2.8 L; 中位2.3 L）较治疗前无明显下降^[11]^。

热消融应用于不可手术切除的NSCLC患者安全性良好。术中常见的不良事件为气胸、咯血，主要发生于穿刺过程。文献报道气胸发生率11%-64%，多数经卧床休息、低流量吸氧等对症治疗后可好转，仅4%-14%的患者需胸腔闭式引流治疗，高龄、伴有慢性阻塞性肺部疾病的患者，气胸风险相对较高^[7-14]^。咯血主要原因为穿刺损伤肺血管和支气管引起，完善术前穿刺计划及加强呼吸训练等措施可有效降低其发生率。Ambrogi等^[14]^报道的一项研究中，咯血发生率约4%。术后常见不良反应为低热（36%），在支持治疗后均可恢复，考虑可能与消融术后局部坏死组织吸收有关^[8]^。另外，少量胸腔积液也较常见，发生率6%-34%，可能与热消融后局部炎性渗出有关，给予支持治疗后可以缓解^[7-10, 14]^。在热消融过程中，目前尚无死亡病例报道。

## 2 热消融在局部晚期NSCLC中的应用

局部晚期NSCLC（Ⅲa期、Ⅲb期），目前权威指南均推荐放化疗为主要治疗方案^[18]^。目前热消融应用于局部晚期NSCLC的文献报道较少。一项国内随机对照研究初步探索了热消融在局部晚期NSCLC中临床疗效，研究组肺内病灶予CT引导下MWA，纵隔淋巴结行放射治疗联合或不联合化疗，对照组给予放射治疗联合或不联合化疗，共98例Ⅲa期或Ⅲb期NSCLC患者被纳入研究，结果显示：两组肺内及纵隔淋巴结病灶的局部控制率未见差异，同样两组2年OS率也无统计学差异（*P*=0.297）^[19]^。局部晚期NSCLC仍然以放化疗为标准治疗，热消融是否可以真正给局部晚期NSCLC患者带来生存获益，目前尚未知，期待将来有更多的研究去探索。

## 3 热消融在晚期NSCLC中的应用

### 3.1 热消融应用于驱动基因阳性、TKI治疗后局限性进展的NSCLC

晚期NSCLC（Ⅳ期）主要以系统治疗为主。表皮生长因子受体（epidermal growth factor receptor, *EGFR*）敏感突变阳性的晚期NSCLC，*EGFR*酪氨酸激酶抑制剂（EGFR-tyrosine kinase inhibitors, EGFR-TKIs）是目前标准一线治疗策略。多项随机对照试验（randomized controlled trial, RCT）研究结果凸显了EGFR-TKIs相对于化疗在*EGFR*敏感突变阳性NSCLC中的优势，为吉非替尼、埃克替尼、厄洛替尼、阿法替尼、达可替尼和奥希替尼等一线治疗晚期NSCLC奠定了基础^[20]^。

表皮生长因子受体酪氨酸激酶抑制剂（epidermal growth factor receptor-tyrosine kinase inhibitors, EGFR-TKIs）治疗终会面临耐药，有研究认为对于局限性进展的患者，在继续原EGFR-TKIs治疗的基础上，同时予局部治疗的干预可能给患者带来更多的临床获益，相反过早停用EGFR-TKIs可能导致疾病的快速进展^[21, 22]^。一项开放性、单臂Ⅱ期研究（ASPIRATIO）结果也支持上述观点，对*EGFR*敏感突变阳性的Ⅳ期NSCLC，在厄洛替尼治疗进展后继续维持厄洛替尼治疗，可得到无进展生存期（progression free survival, PFS）延长3.1个月的生存获益，尤其对于存在19外显子缺失或L585R突变的患者^[23]^。Li等^[24]^对205例*EGFR*敏感突变阳性的晚期NSCLC患者予EGFR-TKI治疗，15例患者出现单个病灶进展后，维持EGFR-TKI治疗，同时进展病灶予MWA治疗，结果中位PFS1、PFS2分别达9.5个月和8个月，中位OS达23个月。Ni等^[25]^探索了MWA在晚期NSCLC非中枢性寡转移中的优势，纳入*EGFR*敏感突变阳性的晚期NSCLC患者予EGFR-TKI治疗，直至出现非中枢性寡转移，实验组在维持EGFR-TKI治疗的基础上对寡转移病灶行MWA治疗，对照组则停用EGFR-TKI，接受细胞毒类药物系统化疗，结果显示：维持EGFR-TKI治疗并联合寡转移灶MWA治疗的患者，中位OS较系统化疗组显著延长（27.7个月*vs* 20.0个月，HR=0.238），中位PFS也明显延长（8.8个月*vs* 5.8个月，HR=0.357）。Wei等^[26]^报道的另外一项研究中，对晚期NSCLC治疗后出现寡转移的患者，辅以微波消融治疗，中位PFS可达14个月。目前，于对棘皮动物微管相关蛋白样4（echinoderm microtubule-associated protein-like 4, EML4）和间变淋巴瘤激酶（anaplastic lymphoma kinase, ALK）和ROS1基因融合等其他靶点阳性晚期NSCLC患者，尚无研究报道TKI联合热消融的治疗模式。

### 3.2 热消融联合化疗应用于驱动基因阴性NSCLC

驱动基因阴性的晚期NSCLC，一线治疗方案仍然以铂类为基础的化疗为主导。国内学者将MWA联合化疗应用于晚期NSCLC，患者经MWA治疗后序惯行铂类为基础的双药化疗，总体客观反应率（overall objective response, ORR）和疾病控制率（disease control rate, DCR）分别可达到28.2%和74.4 %，中位PFS和中位OS分别为8.7个月（95%CI: 5.5-11.9）和21.3个月（95%CI: 17.0-25.4），3级不良反应仅7.9%^[27]^。随后该团队设立了单独化疗为对照组，对比MWA联合化疗与单独化疗的疗效，联合组ORR和肿瘤局部进展时间（time to local progression, TTLP）未显优势（*P*=0.961），但联合治疗组中位PFS显著延长[10.9个月（95%CI: 5.11-16.7）*vs* 4.8个月（95 %CI: 3.9-5.8），*P*=0.001]，中位OS同样较单独化疗显著延长[23.9个月（95%CI: 15.2-32.6）*vs* 17.3个月（95%CI: 15.2-19.3），*P*=0.140]^[28]^。Li等^[29]^开展的另外一项研究得到了类似结果，即化疗联合MWA治疗组的中位PFS及中位OS均显著优于单独化疗组。Du等^[30]^报道的另外一项RFA联合化疗和单独化疗治疗晚期NSCLC的研究显示：联合治疗组2年OS率明显优于单独化疗组（9.31% *vs* 19.9%, *P* < 0.05）。Li等^[31]^在晚期NSCLC一线化疗后，对于部分缓解（partial remission, PR）或稳定（stable disease, SD）的患者，辅以MWA治疗后再次评估，63.3%、24.5%和12.2%分别达到完全缓解（complete response, CR）、PR、SD。

### 3.3 热消融联合免疫治疗在NSCLC中的探索

随着免疫治疗的迅速崛起，为驱动基因阴性的晚期NSCLC带来了新的曙光。Pembrolizumab作为程序性死亡分子1（programmed death-1, PD-1）抑制剂已获批一线治疗驱动基因阴性的晚期NSCLC（PD-L1≥50%）。然而Pembrolizumab对于PD-L1低表达的患者，疗效仍有限。热消融可以使局部肿瘤组织凝固性坏死及肿瘤抗原释放，刺激机体的免疫功能^[32]^。有研究认为热消融与免疫治疗具有一定协同作用，可以增进免疫抑制剂的抑癌作用^[33]^。Shi等^[34]^对结肠癌肝脏转移肿瘤行RFA治疗，结果发现原发肿瘤灶T淋巴细胞浸润增加、PD-L1的表达上调，随后经动物模型研究发现，在RFA治疗初期，促进了肿瘤组织中T淋巴细胞所介导的免疫反应。Schneider等^[35]^首次探讨了RFA对NSCLC免疫功能的影响，在RFA治疗的早期，肿瘤组织中的T淋巴细胞增殖明显，同时激活树突状细胞刺激T细胞表型。然而目前热消融联合PD-1免疫抑制剂的临床研究报道甚少。一项探索热消融联合Nivolumab应用于一线治疗后进展的晚期NSCLC的Ⅱ期临床研究正在进行，期待后期研究结果能给晚期NSCLC带来新的临床治疗策略。

## 4 热消融在NSCLC中面临的挑战

热消融作为局部治疗，同样面临术后复发难题。热消融后复发可分为三种模式，即局部复发、区域复发和远处复发。Beland等^[36]^报道RFA治疗NSCLC，6个月、1年、2年复发率分别为11.3%、32.9%、68.2%，最常见的是局部复发，占38%。对于不可手术切除的Ⅰ期NSCLC，有研究显示：热消融术后6个月、1年、2年复发率分别为4%、6%、22%，但5年复发率高达78%^[8]^。热消融术后复发与肿瘤直径密切相关，多篇研究均认为肿瘤直径 < 2 cm，复发率明显低于直径≥2 cm的患者^[8, 10, 12]^。如何保证消融范围，既可以有效灭活肿瘤，又可以降低术后复发率，显得极为重要。尚待进一步研究精准选择特定患者群体，使热消融给NSCLC患者带来更多的临床获益。未来热消融技术的革新，也将进一步推动其临床应用。

其次，如何把握热消融治疗的适应证是临床较为关注的问题。有合并疾病的患者，是否适合根治性外科手术及如何评判显得至关重要。目前由美国胸科医师协会（American College of Chest Physicians, ACCP）提出的临床实践标准，主要将年龄、肺功能（FEV_1_、DLCO）、左心室功能等作为重要参考指标^[37]^。在ACOSOGZ4033研究中，除参考上述标准外，还将胸外科医师的评估结果作为重要的参考因素^[12]^。纵览近年研究，对于早期不可手术NSCLC的定义不尽相同。其中一些研究中将高龄、心肺功能较差作为不适合手术切除的标准^[11]^。随着外科手术的发展，迫切需要新的、适合目前治疗理念的标准来指导临床决策，以避免超适应证的手术风险。晚期NSCLC，热消融联合EGFR-TKIs、系统化疗的时机和方式，目前尚缺乏规范化。有观点认为联合治疗优于单独治疗，但临床证据级别较低。基于热消融对肿瘤局部微环境的改变，多项研究认为热消融与免疫治疗具有协同作用，可以促进免疫治疗的抗肿瘤治疗。然而多数研究均处于机制研究探索阶段，真正的临床研究数据仍然有限。故热消融联合其他标准治疗应用于晚期NSCLC，同样面临争议和挑战。

热消融治疗后疗效评价也是临床亟待解决的问题。实体肿瘤的疗效评价标准（Response Evaluation Criteria in Solid Tumors, RECIST）以肿瘤最大径为标准。然而热消融治疗后坏死的肿瘤区域仍然存在，同时消融诱发局部炎症反应，CT平扫常表现为肺实质周围模糊片状影，可能需要数月时间才吸收。增强CT扫描可根据病灶强化特征来评价，但热消融后病灶周围组织炎性充血出现的强化表现可能影响CT疗效评价^[38, 39]^。MRI在其他实体肿瘤疗效评估方面较CT理想，但由于肺组织是含气体器官，MRI在肺肿瘤疗效的评估中有一定局限性^[40]^。氟脱氧葡萄糖正电子发射计算机断层扫描术（fluodeoxyglucose-positron emission tomography, FDG-PET）可以通过葡萄糖摄取判断肿瘤活性，较其他影像评估具有明显优势^[41]^。但FDG-PET临床费用昂贵，尤其对于需长期复查随访的患者。在未来新的疗效评价标准或评价技术可以进一步推动热消融的临床应用。

综上，热消融在早期不可手术NSCLC，以及系统治疗后局部进展的晚期NSCLC中具有一定应用前景。如何规范热消融治疗NSCLC的适应证，深入探索其在联合分子靶向治疗、免疫治疗中的临床价值，开发更为理想的疗效评价体系，均是热消融未来探索的方向。目前已有多项关于热消融治疗NSCLC的临床研究正在进行中（表 2），期待其结果提供更多高级别的临床证据来指导临床决策。

**2:** 热消融治疗NSCLC的临床研究 Clinical study of thermal ablation for NSCLC

Study title	Interventions	Phase	Outcome measures	Number enrolled	Time limit		NCT number
Mitochondria-targeted system therapy combined with radiofrequency ablation for early-stage NACLC	Mitochondria-targeted system therapy RFA	NA	DFS, OS	1, 753	2019.01.31 2024.01.31		NCT03840408
Evaluating the safety and efficacy of pembrolizumab combined with MWA for advanced NSCLC	Pembrolizumab MWA	NA	OS, AE, PFS	100	2018.11.01 2020.11.01		NCT03769129
Microwave plus chemotherapy versus chemotherapy for advanced NSCLC	MWA chemotherapy	Phase 3	PFS	275	2015.01 2018.05		NCT02455843
Local ablative therapy for treatment of oligoprogressive, EGFR-mutated, non-small cell lung cancer after treatment with Osimertinib	Osimertinib local ablative	Phase 2	PFS, ORR	100	2016.04.13 2022.09.01		NCT02759835
Microwave ablation in the treatment of stage Ⅰ NSCLC	MWA	Phase 3	OS, DFS	150	2016.09 2019.09		NCT02896166
EGFR: epidermal growth factor receptor; RFA: radiofrequency ablation; DFS: disease free survival; PFS: progression free survival; ORR: objective response rate; OS: overall survival; AE: adverse event.							
